# Central Diabetes Insipidus as a Rare Presentation of Small Cell Lung Cancer: A Case Report

**DOI:** 10.7759/cureus.114983

**Published:** 2026-08-22

**Authors:** Cole M Lamanteer, Eric Prater, Michael Phy

**Affiliations:** 1 Department of Internal Medicine, Texas Tech University Health Sciences Center, Lubbock, USA

**Keywords:** central diabetes insipidus (cdi), hypernatremia, paraneoplastic syndromes, pituitary metastasis, small cell lung cancer (sclc)

## Abstract

Small cell lung carcinoma (SCLC) is an aggressive neuroendocrine malignancy commonly associated with paraneoplastic syndromes, most often the syndrome of inappropriate antidiuretic hormone secretion (SIADH). Central diabetes insipidus (CDI) is a rare manifestation and may result from hypothalamic-pituitary involvement by metastatic disease. A 53-year-old man with a significant smoking history presented with abdominal pain and was found to have a right perihilar lung mass with extensive metastatic disease. Liver biopsy confirmed SCLC. During hospitalization, he developed acute polyuria with worsening hypernatremia, elevated serum osmolality, and inappropriately dilute urine. A marked concentrating response following desmopressin administration supported the diagnosis of CDI, while MRI of the brain demonstrated a sellar-suprasellar mass with laboratory findings concerning for central hypothyroidism, indicating hypothalamic-pituitary axis disruption. Given the rapid onset of symptoms and widespread metastatic disease, pituitary metastasis was considered the most likely etiology. Despite initiation of desmopressin and systemic chemotherapy, the patient experienced progressive clinical decline and ultimately transitioned to comfort care. This case highlights a rare presentation of SCLC with CDI likely due to pituitary metastasis. New-onset polyuria and hypernatremia in patients with advanced malignancy should prompt evaluation for CDI to allow timely diagnosis and management, even in the setting of poor overall prognosis.

## Introduction

Diabetes insipidus (DI) is a rare endocrine disorder that is characterized by impaired vasopressin secretion (central DI, CDI), or renal resistance to vasopressin (nephrogenic DI), with resultant polyuria and polydipsia [1]. CDI results from deficient secretion of arginine vasopressin (AVP) due to hypothalamic-pituitary injury, causing impaired renal water reabsorption and large volumes of dilute urine. In contrast, syndrome of inappropriate antidiuretic hormone secretion (SIADH) is a well-recognized paraneoplastic manifestation of SCLC resulting from inappropriate AVP activity, leading to water retention and typically hyponatremia. This opposing pathophysiology highlights the unusual nature of CDI in the setting of SCLC. The posterior pituitary, having direct arterial supply from the inferior hypophyseal artery originating from the internal carotid artery, is particularly vulnerable to metastatic disease [2].

CDI is most often caused by lesions of the pituitary or hypothalamus, which can include metastases, although traumatic and autoimmune destruction of hormone-synthesizing neurons is also possible. The most common neoplastic lesions causing CDI are craniopharyngiomas, germinomas, as well as metastases from the lung and breast; pituitary adenomas rarely present with CDI [3]. In patients with known or suspected malignancy, the presence of new-onset DI is an uncommon finding that should prompt evaluation for pituitary metastases.

Pituitary metastasis is rare, accounting for about 1% of surgically treated pituitary lesions and 0.4-1% of intracranial metastases [3,4]. Lung and breast cancers are the most common primary sources of pituitary metastasis, making evaluation for hypothalamic-pituitary axis endocrine abnormalities in the setting of this malignancy an important part of management [5]. Additionally, DI is an atypical manifestation of small cell lung carcinoma (SCLC), most often reflecting metastatic involvement of the hypothalamic-pituitary axis rather than a true paraneoplastic syndrome.

## Case presentation

A 53-year-old man with a history of hypertension, chronic alcohol use, and a >30 pack-year smoking history presented with abdominal pain and constipation for six weeks. He had been evaluated at an outside facility the day prior, where CT imaging of the abdomen and pelvis demonstrated multiple intra-abdominal and thoracic lesions concerning for malignancy, as well as splenic vein thrombosis. He presented to our institution for further evaluation and management. He reported progressive abdominal distention, decreased appetite, and night sweats, but denied weight loss, nausea, vomiting, melena, or hematochezia. His last bowel movement was two days prior and described as small and thin. He also endorsed mild shortness of breath, which he attributed to abdominal distention. He had no prior history of endoscopic evaluation and did not have an established primary care provider.

At presentation in the emergency department, the patient was noted to be hypertensive with otherwise normal temperature, heart rate, and oxygen saturation (Table 1). Physical examination revealed a firm, distended abdomen with diffuse tenderness, most prominent in the epigastric region, and normoactive bowel sounds. Respiratory examination demonstrated mild bibasilar wheezing, while cardiovascular, neurologic, musculoskeletal, and mental status examinations were unremarkable. Laboratory evaluation was notable for leukocytosis, hypernatremia, hypokalemia, and mild transaminitis (Table 2).

**Table 1:** Vital signs at presentation in the emergency department SpO_2_: peripheral oxygen saturation

Vital sign	Measurement	Reference Range
Blood pressure (systolic/diastolic)	171/116 mmHg	90/60 to 120/80 mmHg
Temperature	98.4 F	97 F to 99 F
Heart rate	72	60 to 100 beats per minute
SpO_2_	94% on a 2/L/min nasal cannula	95%-100% on room air

**Table 2:** Notable laboratory findings in the emergency department WBC: white blood cell; ALT: alanine aminotransferase; AST: aspartate aminotransferase

Lab	Value	Reference Range
WBC (×10³/µL)	14.48	4.0-10.0
Serum sodium (mmol/L)	147	135-145
Serum potassium (mmol/L)	2.6	3.5-5.5
ALT (U/L)	69	5-41
AST (U/L)	50	5-37

**Figure 1:**
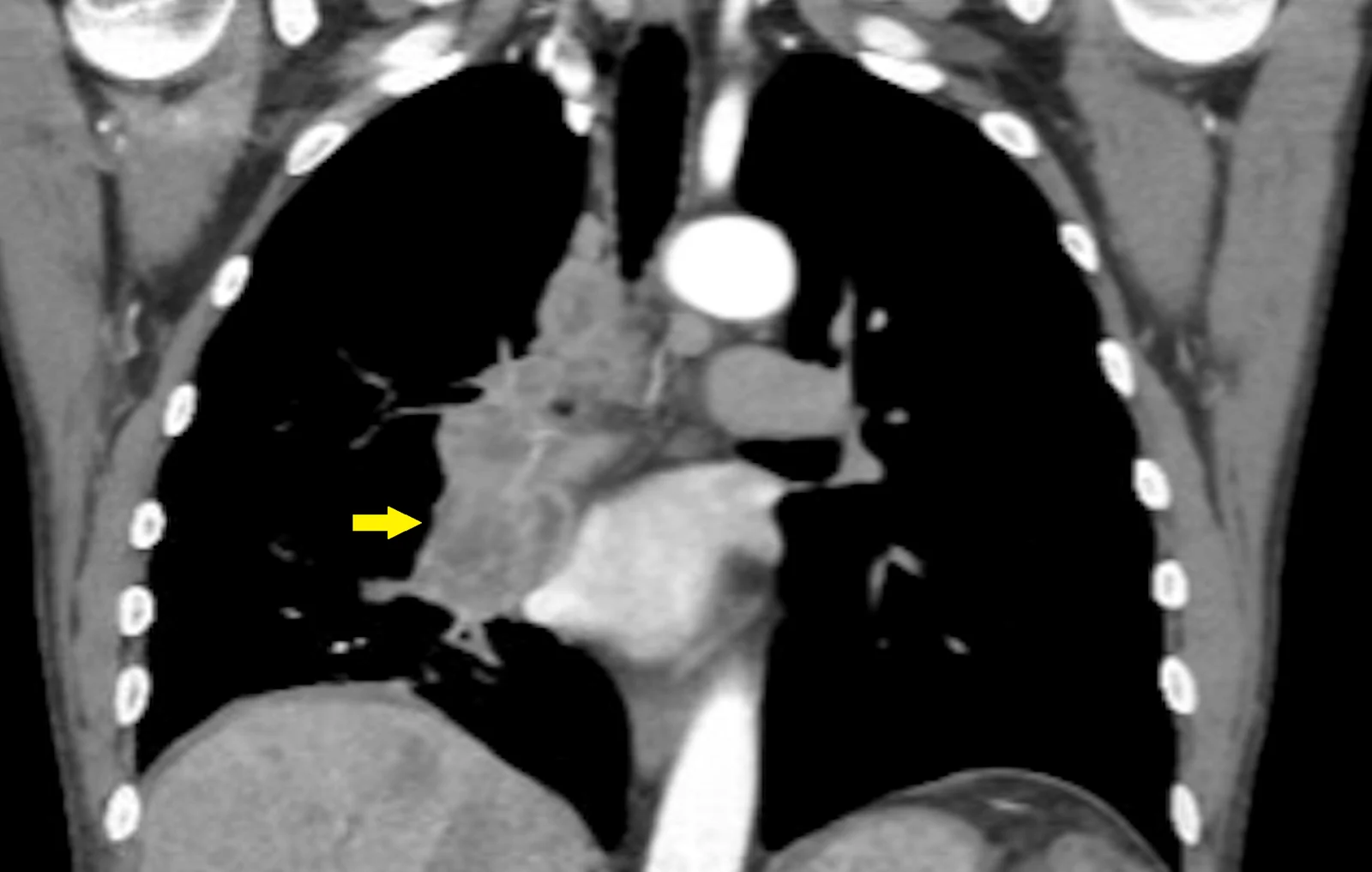
CT scan with IV contrast of the chest demonstrating a right perihilar mass The yellow arrow is pointing towards the 4.7 x 3.1 cm cystic and necrotic right perihilar mass.

**Figure 2:**
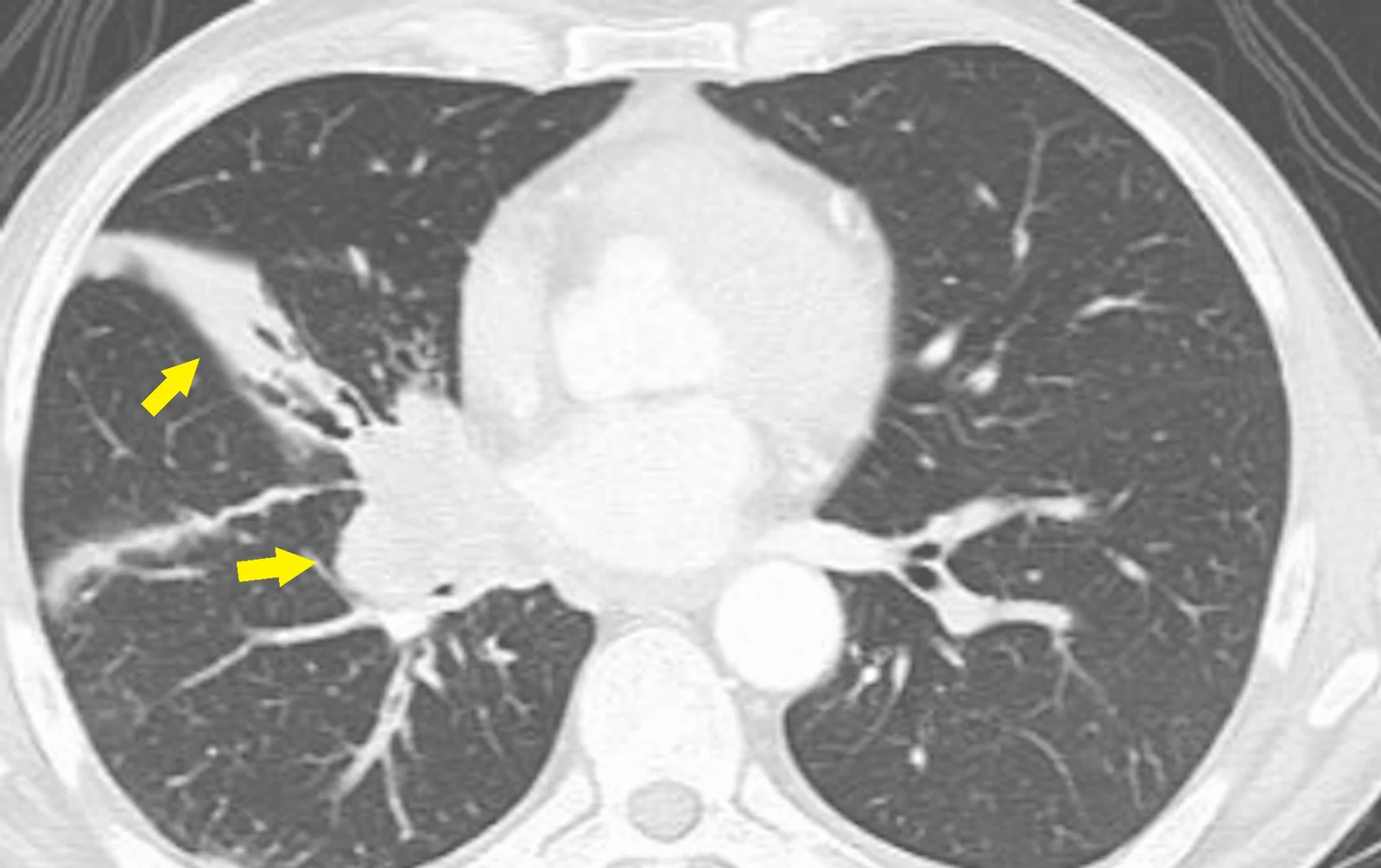
CT scan with IV contrast of the chest showing a right perihilar mass with associated bronchial obstruction and atelectasis The top yellow arrow indicates a hyperdense area of atelectasis in association with the obstructive bronchial mass indicated by the bottom arrow.

**Figure 3:**
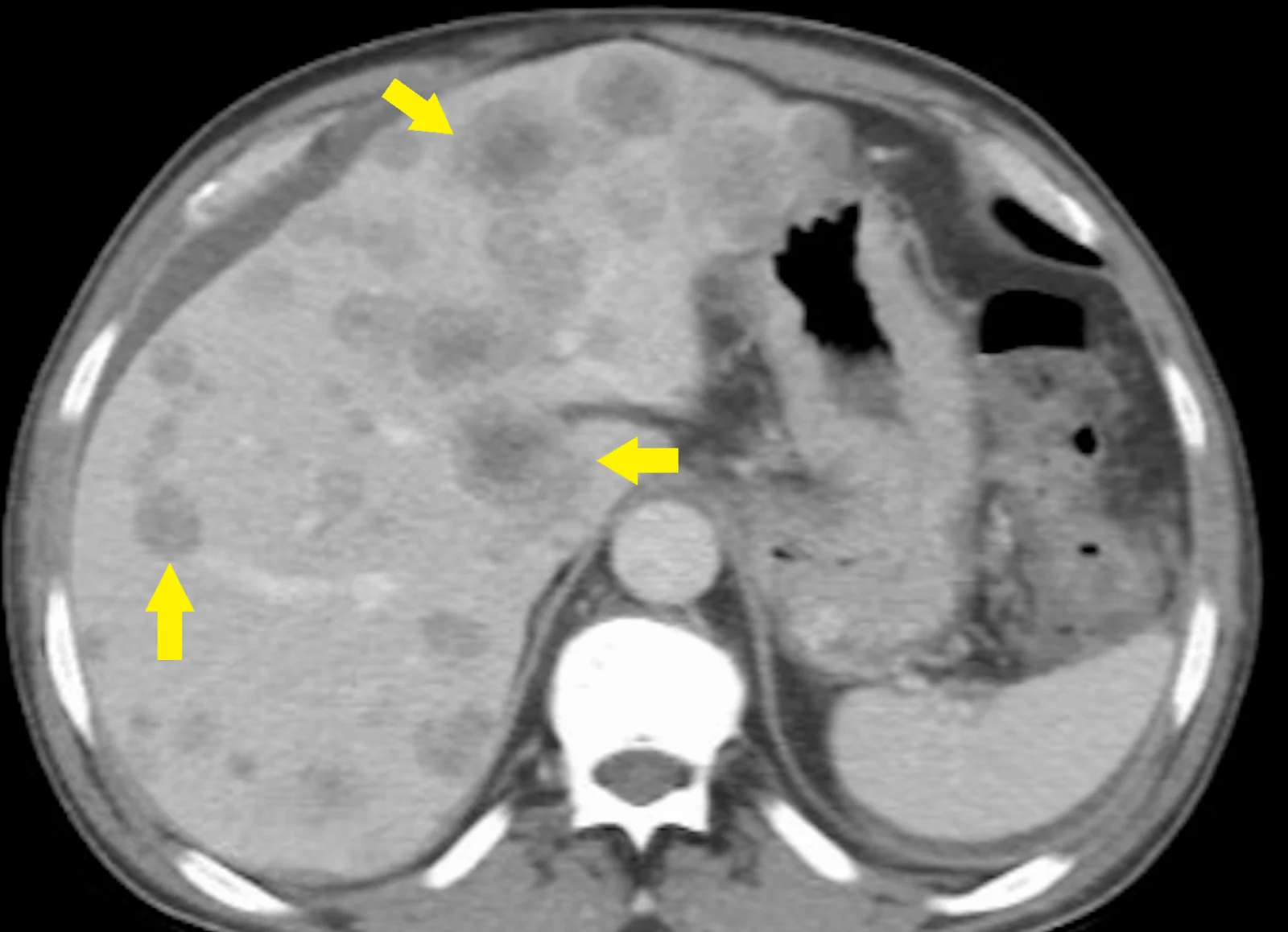
CT scan of the abdomen demonstrating numerous hepatic metastases The yellow arrows indicate representative hypodense hepatic lesions consistent with metastases.

**Figure 4:**
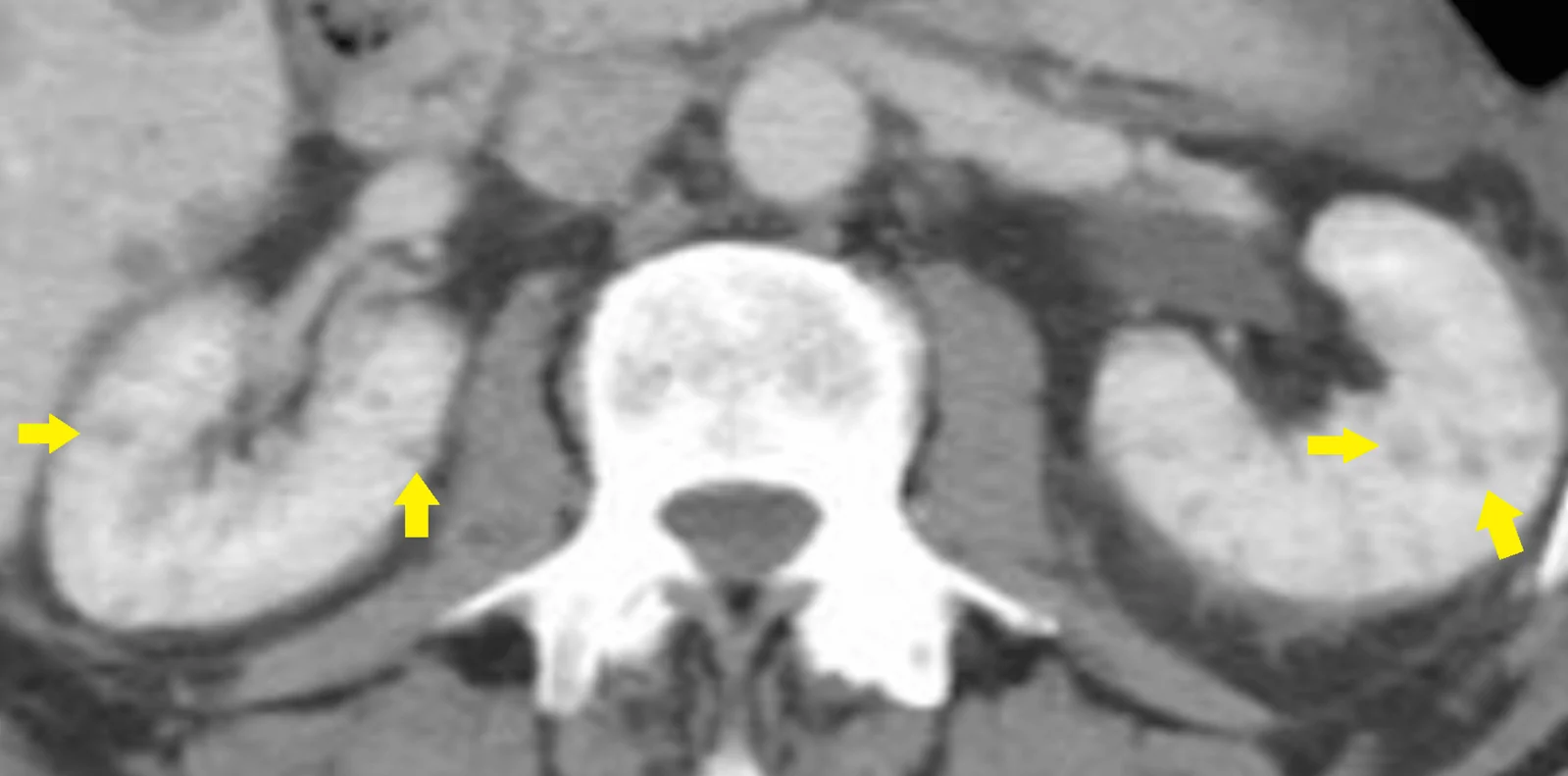
CT scan of the abdomen demonstrating multiple bilateral renal cortical hypodensities The yellow arrows point to multiple bilateral renal cortical hypodensities.

**Figure 5:**
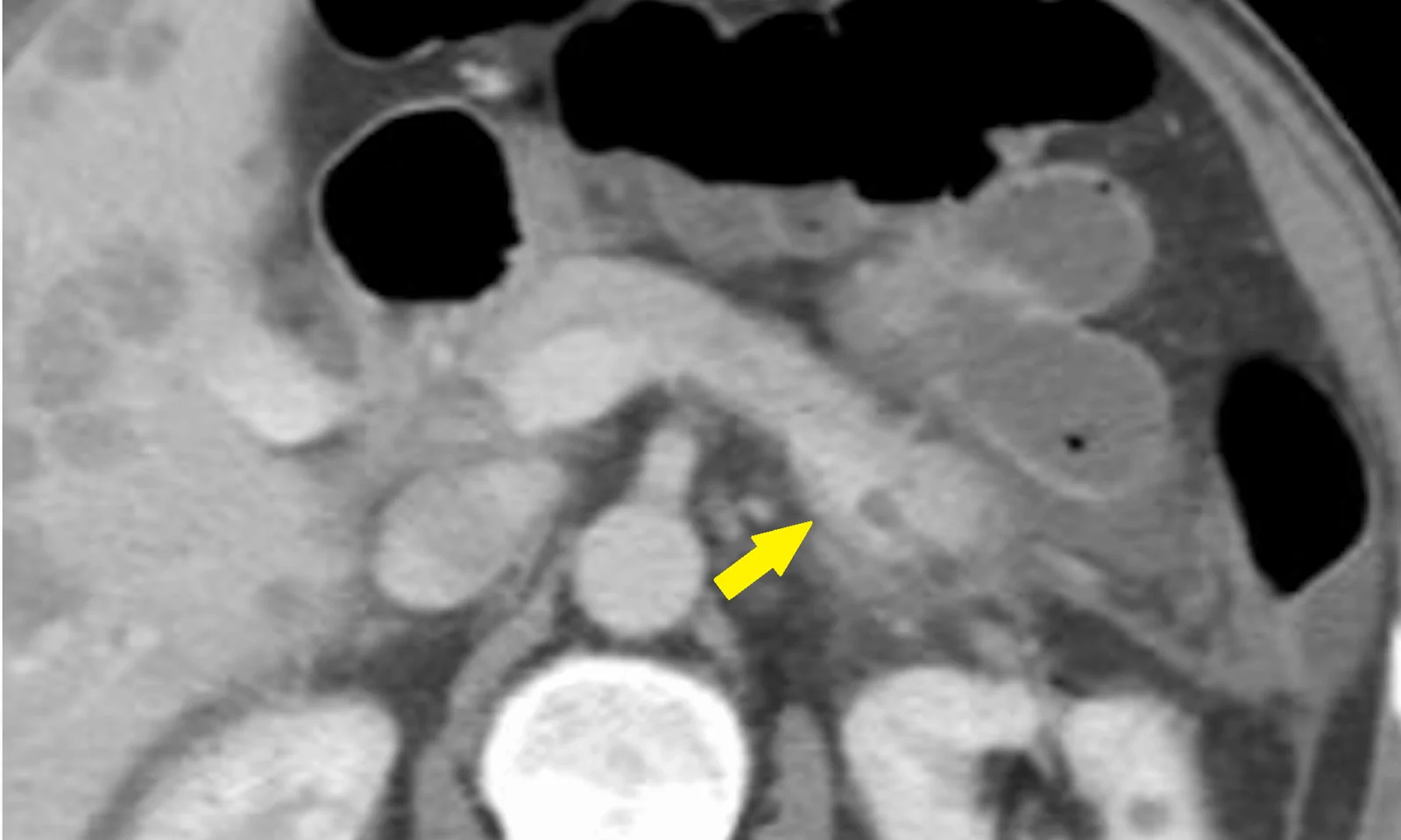
CT scan of the abdomen demonstrating splenic vein thrombosis Thrombosis of the splenic vein indicated by the yellow arrow.

**Figure 6:**
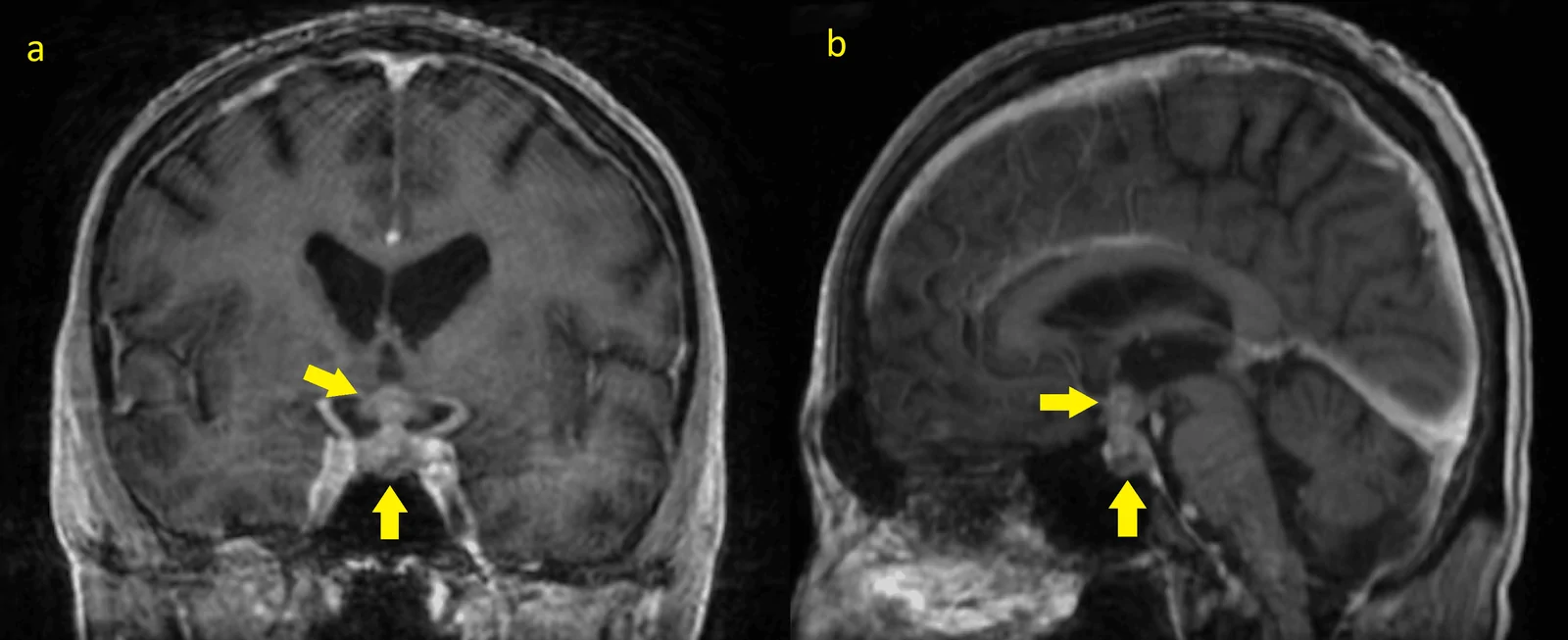
MRI of the brain with contrast demonstrating the bilobed sellar-suprasellar mass The yellow arrows point to each half of the bilobed appearing structure as seen in both (a) coronal and (b) sagittal views.

Imaging studies revealed a 4.7 × 3.1 cm cystic and necrotic right perihilar mass with associated bronchial obstruction and atelectasis, as well as widespread metastatic disease, including multiple hepatic lesions and bilateral renal cortical hypodensities (Figures 1-4). CT imaging also demonstrated splenic vein thrombosis (Figure 5). MRI of the brain with contrast demonstrated a bilobed enhancing sellar-suprasellar mass extending into the retrochiasmatic and hypothalamic regions, with differential considerations, including metastasis, embolism, and pituitary macroadenoma (Figure 6). A CT-guided liver biopsy confirmed a high-grade neuroendocrine tumor consistent with small cell lung carcinoma.

During hospitalization, the patient developed significant polyuria, with urine output up to 4.5 L per day in the absence of diuretic therapy. Laboratory evaluation demonstrated an elevated serum sodium and serum osmolality with inappropriately low urine osmolality (Table 3). Fluid intake and subjective polydipsia were not clearly documented in the available medical record. Given concern for DI, 4 micrograms of intravenous desmopressin was administered. Following desmopressin administration, laboratory results the next day showed urine osmolality increasing from 109 to 473 mOsm/kg, while subsequent urine output decreased from 4.5 to 1.97 L/day. Serum sodium subsequently decreased from 159 to 144 mmol/L (Table 4). These findings supported the diagnosis of CDI. Additional endocrine evaluation revealed a low free T4 with inappropriately low-normal TSH consistent with possible central hypothyroidism in the setting of hypothalamic-pituitary axis involvement.

**Table 3:** Lab values prior to desmopressin administration T4: thyroxine; TSH: thyroid-stimulating hormone

Lab	Value	Reference Range
Serum sodium (mmol/L)	159	135-145
Serum osmolality (mOsm/kg)	323	275-295
Urine osmolality (mOsm/kg)	109	>300
Urine output (L/day)	4.5	0.8-2.0
Free T4 (ng/dL)	0.70	0.8-1.8
TSH (mIU/L)	0.39	0.4-4.0

**Table 4:** Lab values after desmopressin administration

Lab	Value	Reference Range
Serum sodium (mmol/L)	144	135-145
Serum osmolality (mOsm/kg)	296	275-295
Urine osmolality (mOsm/kg)	473	>300
Urine output (L/day)	1.97	0.8-2.0

Following the response to desmopressin supporting the diagnosis of CDI, scheduled desmopressin was initiated to manage hypernatremia. Repeat thyroid function tests were ordered with planned follow-up in the outpatient setting for endocrine evaluation of possible central hypothyroidism.

The patient was placed on anticoagulation with intravenous heparin for the splenic vein thrombus. An oncology consultation was obtained for treatment planning of SCLC. A chemo-port was placed. The first cycle of chemotherapy with carboplatin, etoposide, and atezolizumab was initiated for management of SCLC and extensive metastatic disease. Neurology was consulted for possible embolic etiology of the suprasellar mass, and a transthoracic echocardiogram (TTE) with agitated saline contrast failed to show right-to-left shunting. A transesophageal echocardiogram (TEE) was then recommended to rule out cardiac mural thrombus but was deferred in the setting of ongoing anticoagulation.

At this time, the patient was discharged in stable condition on desmopressin 0.1 mg PO, spironolactone 50 mg PO BID, and PO potassium chloride 20 mEq for hypokalemia, as well as rivaroxaban, with multiple planned outpatient follow-up visits, most significantly with neurosurgery and radiation oncology pending results of a scheduled MRI of the brain.

Ten days later, the patient returned to the emergency department sent from the outpatient oncology clinic for severe abdominal pain and intermittent chest pain. Upon workup, he was found to be hypotensive with severe electrolyte abnormalities and subsequently admitted to the medical ICU. After improvement of hypotension and correction of electrolyte imbalances, he was transferred to the hospital floor. During the next three weeks, he developed pancreatitis, fungemia, and progressive decline, and palliative care was consulted to discuss goals of care. The patient's family members elected for comfort care measures, and the patient passed three days later.

## Discussion

SCLC is strongly associated with paraneoplastic endocrine syndromes, most commonly SIADH [6]. In contrast, CDI is a rare manifestation and reflects a fundamentally different mechanism, making prompt recognition critical for timely diagnosis and management [6,7].

This patient's development of large-volume urine output in the absence of diuretic therapy prompted investigation for DI. The presence of polyuria (> 3 L/day of dilute urine) with the inability to concentrate urine and resultant hypernatremia were clinical signs supporting this diagnosis. Serum osmolality was elevated at 323 mOsm/kg with a dilute urine osmolality of 109 mOsm/kg and serum sodium of 159 mmol/L. Desmopressin was administered to differentiate central from nephrogenic DI, as management differs substantially [8]. Following desmopressin administration, urine osmolality increased from 109 to 473 mOsm/kg (a 364 mOsm/kg, or approximately 334%, increase) while urine output subsequently decreased from 4.5 to 1.97 L/day. This marked concentrating response supported a central rather than nephrogenic etiology. In less definitive presentations, a supervised water deprivation test may be used to assess urinary concentrating ability and help differentiate central DI, nephrogenic DI, and primary polydipsia [8]. During water deprivation, failure to appropriately concentrate the urine supports DI, while the subsequent response to desmopressin aids in distinguishing central from nephrogenic DI. A formal water deprivation test was not performed in this patient given the presence of marked hypernatremia, elevated serum osmolality, and inappropriately dilute urine at the time of evaluation.

Neuroimaging in this case supported a structural etiology for CDI. MRI demonstrated a bilobed enhancing sellar-suprasellar mass extending into the retrochiasmatic and hypothalamic regions, with differential considerations, including metastasis, embolism, and pituitary macroadenoma. Although imaging characteristics of pituitary metastases can overlap with those of primary pituitary tumors such as macroadenomas [2], features including rapid symptom onset, hypothalamic involvement, and widespread metastatic disease favored metastatic involvement [3,4]. Although tissue confirmation of the sellar-suprasellar lesion was not obtained, metastasis was considered the leading etiology in the setting of biopsy-confirmed SCLC and disseminated disease. Nevertheless, the absence of histopathologic confirmation precludes definitive attribution of the sellar-suprasellar lesion to pituitary metastasis.

From a prognostic standpoint, pituitary metastasis typically occurs in the setting of advanced, disseminated malignancy and is associated with poor outcomes [4,5]. The rapid clinical decline observed in this patient is consistent with the aggressive nature of extensive-stage SCLC [7]. In this context, the development of CDI may represent an endocrine manifestation of advanced central nervous system involvement [3,4]. Accordingly, management of CDI is primarily supportive, with desmopressin effectively correcting free water losses and hypernatremia [1,8], though it does not address the underlying malignancy.

## Conclusions

This case highlights an uncommon but clinically significant presentation of SCLC with CDI in the setting of hypothalamic-pituitary axis disruption from a space-occupying lesion. In a patient with newly diagnosed metastatic SCLC and rapid-onset polyuria, hypernatremia, and low urine osmolality, quick recognition of DI and an observed responsiveness to desmopressin was essential in diagnosing CDI.

The presence of widespread metastatic disease and the tendency for SCLC to involve the central nervous system made pituitary metastasis the leading consideration for the suprasellar lesion and associated CDI. This case underscores several clinical lessons. DI is a rare but important manifestation of SCLC, distinct from the more commonly associated SIADH. Additionally, new-onset polyuria and hypernatremia in patients with advanced malignancy should prompt evaluation for CDI. Early recognition of these manifestations may guide timely diagnostic evaluation and supportive management, even in the context of a poor overall prognosis.
